# Teleost microbiomes: the state of the art in their characterization, manipulation and importance in aquaculture and fisheries

**DOI:** 10.3389/fmicb.2014.00207

**Published:** 2014-06-02

**Authors:** Martin S. Llewellyn, Sébastien Boutin, Seyed Hossein Hoseinifar, Nicolas Derome

**Affiliations:** ^1^Département de Biologie, Institut de Biologie Intégrative et des Systèmes, Université LavalQuébec, QC, Canada; ^2^Molecular Ecology and Fisheries Genetics Laboratory, School of Biological Sciences, University of WalesBangor, UK; ^3^Department of Fisheries, Gorgan University of Agricultural Sciences and Natural ResourcesGorgan, Iran

**Keywords:** fish, microbiota, probiotics, aquaculture, fisheries

## Abstract

Indigenous microbiota play a critical role in the lives of their vertebrate hosts. In human and mouse models it is increasingly clear that innate and adaptive immunity develop in close concert with the commensal microbiome. Furthermore, several aspects of digestion and nutrient metabolism are governed by intestinal microbiota. Research on teleosts has responded relatively slowly to the introduction of massively parallel sequencing procedures in microbiomics. Nonetheless, progress has been made in biotic and gnotobiotic zebrafish models, defining a core microbiome and describing its role in development. However, microbiome research in other teleost species, especially those important from an aquaculture perspective, has been relatively slow. In this review, we examine progress in teleost microbiome research to date. We discuss teleost microbiomes in health and disease, microbiome ontogeny, prospects for successful microbiome manipulation (especially in an aquaculture setting) and attempt to identify important future research themes. We predict an explosion in research in this sector in line with the increasing global demand for fish protein, and the need to find sustainable approaches to improve aquaculture yield. The reduced cost and increasing ease of next generation sequencing technologies provides the technological backing, and the next 10 years will be an exciting time for teleost microbiome research.

## Introduction

The bacteria that colonize the internal and external epidermal surfaces of metazoans are thought to outnumber their host cells by at least 10 to 1 (Human Microbiome Project, 2012). Adult humans contain over a kilogram of such organisms (Ley et al., 2008; Human Microbiome Project, 2012; Karlsson et al., 2013). The emergence and evolution of metazoan organisms has undoubtedly involved close partnership with bacterial life. As such, the relationship that exists between vertebrates and their bacterial colonists dates back hundreds of millions of years (Ley et al., 2008). The microbial metagenome dwarfs that of their hosts (Qin et al., 2010). Numerous metabolic processes vital for host fitness and survival may be assigned to, or facilitated by, their microbial community.

Definition of the services provided by a host microbiome depends on our ability to establish its composition and functional capacity. Furthermore, functional stability in space and time may provide clues to recruitment and host fitness constraints on community structure (Costello et al., 2009; Turnbaugh et al., 2009a). Next-generation sequencing techniques, including amplicon and shot-gun approaches, and associated bioinformatic tools have revolutionized our ability to count and classify commensal bacteria. Concurrently, DNA database development for reliable classification of taxonomy (e.g., GreenGenes, Silva), and functionality (e.g., UniProt, Swiss-prot) has facilitated data interpretation. Large-scale multi-partner projects, particularly the Human Microbiome Project (2012), have driven much of the tool development in this area and are also responsible for the instigation of standard operating procedures to facilitate comparisons between samples, centers, and studies. As such, sophisticated hypotheses across large and dispersed cohorts of individuals can be addressed including the impacts of lifestyle, (e.g., Turnbaugh et al., 2006), disease (Morgan et al., 2012), and antibiotic treatment (Perez-Cobas et al., 2013). Studies frequently document perturbations in meta-community structure that accompany these phenomena as well as perturbations that may have a predictive value for certain metabolic diseases (collectively called dysbiosis) (Karlsson et al., 2013). More important still is to establish a causal link between dysbiosis (imbalance in the microbiome) and pathology. In proving causality, “forward microbiomics” are highly attractive (introducing artificial or transplanting microbiomes into naïve hosts). Humanized germ free (gnotobiotic) mouse models, transplanted with human fecal microbiomes, have corroborated dietary microbiome shifts observed in the clinic (Turnbaugh et al., 2009b). Furthermore, transplantation of “obese” human microbiomes into germ-free animals can modulate mouse metabolism toward adiposity and increased body mass (Ridaura et al., 2013).

**Figure 1:**
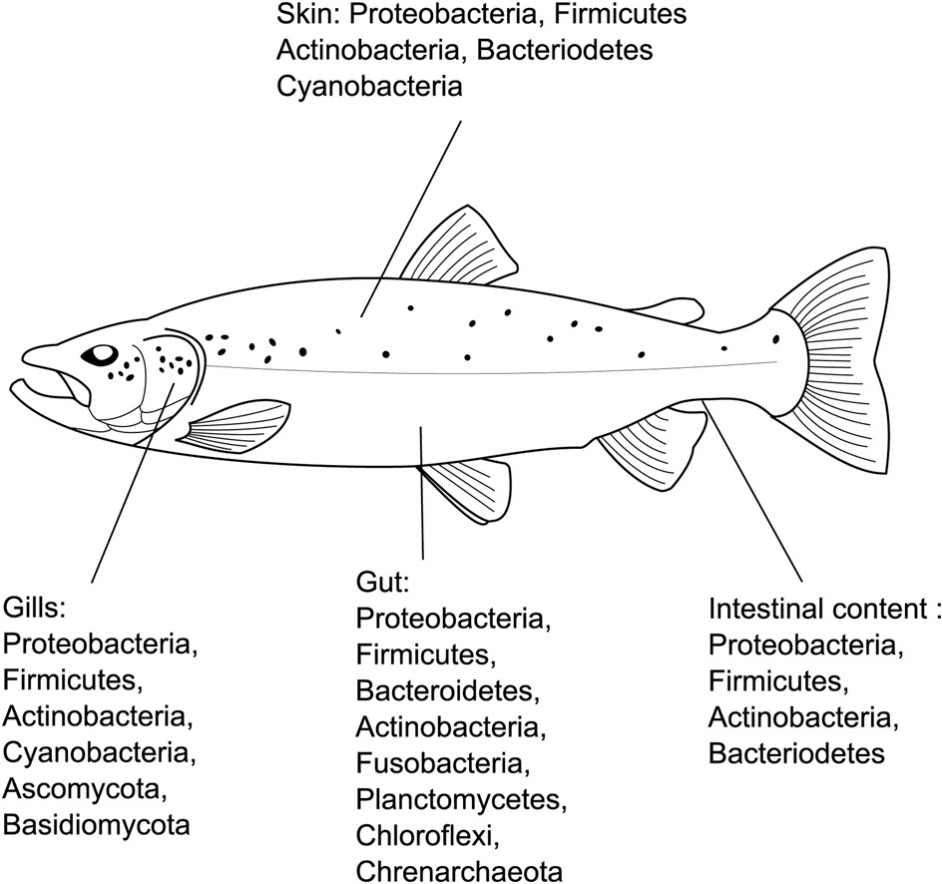
**General microbiological findings on fish microbiota**. This overview synthesizes the major phyla present in the different organs of fish from different species. Bacterial phyla included are correspond to those which made up >80% of sequences characterized from a given tissue/organ in each study. Only studies that employed direct sequencing (clone libraries/amplico-seq) are included.

Teleost microbiome research lags well behind that in humans and mouse models. Nonetheless, thanks in part to the efforts of Rawls and collaborators, the nature of the Zebra fish gut microbiome was established relatively early in the meta-sequencing goldrush. Their work revealed fascinating reciprocal differences between mammalian and teleost microbiota, as well as the first gnotobiotic teleost model (Rawls et al., 2006). Later studies revealed a “core microbiome” among this species, dominated by γ-Proteobacteria and enriched with a diverse assemblage of Fusobacteria species (Roeselers et al., 2011). Importantly, striking similarities were observed between the microbiomes of domesticated and wild individuals, implying a role for host selection on microbiota, and to an extent validating the conclusions of previous laboratory studies. As well as *D. reria*, several other teleost species have had their microbiota scrutinized via either culture dependent or independent techniques. Studies conducted to date, the tools used and species examined, are summarized in Table 1, and a broad overview of their rather incomplete findings in Figure 1. Unsurprisingly the focus has been aquaculture species, although some wild individuals have also been studied. Overall there has been important progress in recent years, albeit uncoordinated and sporadic.

**Table 1:** **Studies evaluating the diversity of teleost-associated microbial communities**.

**Study**	**Fish species**	**Fish origin^a^**	**Organ**	**Sequences derived (approx.)**	**Target/technique**	**Phyla (order of abundance)**	**Notable genera/findings**
Di Maiuta et al., 2013	Panaque sp. (catfish)	A	Faeces samples externally	143,670	16S/454 pyroseqeuncing	Fusobacteria, Cyanobacteria, Beta-proteobacteria, Flavobacteria, Clostridia + other minor groups	Putative cellulolytic bacteria identified Aeromonas sp., Flavobacterium sp., Bacteroides sp., Clostridium sp., and Pseudomonas sp.
Desai et al., 2012	*Oncorhynchus mykiss*	A	Intestinal contents	99,568	16S/454 pyroseqeuncing + DGGE	Proteobacteria, Firmicutes, Actinobacteria, Bacteriodetes	NA
Ye et al., 2014	*Dorosoma cepedianum*	W	Intestinal mucosa and contents	400,000+	16S/454 pyroseqeuncing	Cyanobacteria/Cholorplast, Proteobacteria, Actinobacteria, Firmicutes, Bacteriodetes, Fusobatceria, Planctomycetes, Chloroflexi, Crenarchaeota	Significant differences between foregut and hindgut microbiota, but not between species
Ye et al., 2014	*Hypophthalmichthys molitrix*	W	Intestinal mucosa and contents	400,000+	16S/454 pyroseqeuncing	Cyanobacteria/Chloroplast, Proteobacteria, Actinobacteria, Firmicutes, Bacteriodetes, Fusobatceria, Planctomycetes, Chloroflexi, Crenarchaeota	Significant differences between foregut and hindgut microbiota, but not between species
Geraylou et al., 2013	*Acipenser baerii*	A	Hindgut contents	29,318	16S/454 pyroseqeuncing	Fusobacteria/Firmicutes, Chlamydiae, Bacteriodetes, Actinobacteria	Arabinoxylan oligosaccharide prebuiotics modulate hindgut microbiome composition
Star et al., 2013	*Gadus morhua*	W	Intestinal contents	280,447	16S/454 pyroseqeuncing	Proteobacteria (mostly Vibrionacae), Bacteriodetes, Firmicutes, other minor groups	Large inter-indivudual differences in community composition for fish captured at the same site
Li et al., 2013	*Cyprinus carpio* (transgenic)	A	Intestinal mucosa and contents	621,110	16S/454 pyroseqeuncing + DGGE	Proteobacteria, Fusobacteria, Bacteroidetes, Firmicutes	Differential abundance of bacterial phyla between fast growing transgenic and wild type. Firmicutes: Bacteriodetes ratio differences between transgenic and wild type
Semova et al., 2012	*Dario rerio*	A	Hindgut	10,000+ (data not shown)	16S/454 Pyrosequencing	Firmicutes, Proteobatceria, Bacteriodetes + minor phyla	Microfolora enhance fatty acid uptake in the zebrafish intestine
Wu et al., 2012b	*Ctenopharyngodon idellus*	A	Intestinal mucosa	93,991	16S/454 Pyrosequencing	Firmicutes, Baceriodes, Proteobatceria, Spirochaetes	Cellulose digesting genera present—Anoxybacillus, Leuconostoc, Clostridium, Actinomyces, Citrobacter
Wu et al., 2012b	*Ctenopharyngodon idellus*	A	Intestinal contents	93,991	16S/454 Pyrosequencing	Firmicutes, Cyanobacteria, Proteobacteria, Bacteriodetes	Cellulose digesting genera present—Anoxybacillus, Leuconostoc, Clostridium, Actinomyces, Citrobacter
Roeselers et al., 2011	*Dario rerio*	A/Wild	Intestinal mucosa and contents	22,980	16S/454 Pyrosequencing, Sanger sequence, TRFLP profiling	Proteobacteria, Fusobacteria, Firmicutes, Actinobateria	Core microbiome: γ-Proteobacteria, β-Proteobacteria, Fusobacteria, Bacilli, Flavobacteria, Actinobacteria classes, Aeromonas, Shewanella
Martin-Antonio et al., 2007	*Solea senegalensis*	A	Intestinal mucosa and contents	176	16S/Culture + Sanger Sequencing	alpha-proteobacteria, gamma-proteobacteria, firmicutes	Temperature and diet both influence microbiota present
Sun et al., 2009	*Epinephelus coioides*	A	Intestinal contents	17	16S/Culture + Sanger Sequencing	Beta -proteobacteria, Gamma-proteobacteria, Firmicutes	Species unequally dispersed beween fast and slow growing phenotypes (e.g., *Bacillus pumilis* super-abundant in fast growers)
Huber et al., 2004	*Oncorhynchus mykiss*	A	Intestinal contents	146	16S/Culture + Sanger Sequencing	Beta -proteobacteria, Gamma-proteobacteria	DAPI staining and FISH analysis demoastrate large number of unculturable bacterial species present
Skrodenyte-Arbaciauskiene et al., 2008	*Salmo salar* (juvenile, freshwater)	W	Intestinal contents	52	16S/Culture + Sanger Sequencing	Gamma-proteobacteria, firmicutes	Principal differences were present between *S. trutta* and *S. salmo* were at bacterial species level
Skrodenyte-Arbaciauskiene et al., 2008	*Salmo trutta* (juvenile, freshwater)	W	Intestinal contents	47	16S/Culture + Sanger Sequencing	Gamma-proteobacteria	Principal differences were present between *S. trutta* and *S. salmo* were at bacterial species level
Skrodenyte-Arbaciauskiene et al., 2006	*Salmo trutta* fario	W	Intestinal contents	100	16S/Culture + Sanger Sequencing	Gamma-proteobacteria	Multiple differences at family and species level between populations isolated from two different river systems
Wu et al., 2012a	*Pelteobagrus fulvidraco*	A	Midgut contents, midgut mucus	74	16S/Culture + Sanger Sequencing	Firmicutes, Proteobacteria, Bacteriodetes, Fusobacteria	Different bacterial genera between gut contents and mucosa. Stomach contents conatained Chloroflexi, while mucous Actinobacteria
Cantas et al., 2011	*Salmo salar* (juvenile)	A	Intestinal mucosa and contents	18	16S/Culture + Sanger Sequencing	Gamma-proteobacteria, firmicutes, actinobacteria	Differences between dilpoid and triploid individuals non-significant
Valdenegro-Vega et al., 2013	*Thunnus maccoyii*	Ranched	Gills, Spleen, Kidneys	24	16S/Culture + Sanger Sequencing	(no order) *Vibrio* and *Photobacterium* sp. predominate	–
Cantas et al., 2012	*Dario rerio*	A	Intestinal contents	13	16S/Culture + Sanger Sequencing	Gamma-proteobacteria, beta-proteobatceria, alpha-proteobatceria, firmicutes	–
Tetlock et al., 2012	*Petromyzon marinus*	A	Intestinal contents	682	16S/DGGE + Sanger sequencing	Proteobacteria	Dominated by Aeromonas species
Shiina et al., 2006	*Takifugu niphobles*	W	Intestinal contents	24	16S/DGGE + Sanger sequencing	Firmicutes, Gamma-proteobacteria, Spirochaetes	Cultivable species restricted in greater part to Vibrio species
Tetlock et al., 2012	*Petromyzon marinus*	A	Intestinal mucosa and contents	682	16S/DGGE + Sanger sequencing	Proteobacteria, Bacteriodetes, Tenericutes, + minor phyla	Hugely diverse environment, multiple genera and species
He et al., 2010	*Oreochromis* sp.	A	Intestinal contents	19	16S/DGGE + Sanger sequencing	Cyanobacteria. Proteobacteria, Firmicutes, Actinobacteria, Fusobacterium	Significant influence on antibiotics on gut microbiota
Silva et al., 2011	*Carassius auratus*	A	Intestinal mucosa and contents	60	16S/DGGE + Culture + Sanger Sequencing	Gamma-proteobacteria, Firmicutes	Dominated by Aeromonas species
Svanevik and Lunestad, 2011	*Scomber scombrus*	W	Gills/skin/inestine contents	99	16S/DGGE + Culture + Sanger Sequencing	Gamma-proteobacteria, Firmicutes	Vibrio, Pscrobatcer immobilis, Oceanisphaera and some Shewanella species only present in the gut (samples direct from purse seine onlu included)
Kühlwein et al., 2013	*Cyprinus carpio* L.	A	Intestinal contents	27	16S/DGGE + Culture + Sanger Sequencing	(no order) Proteobacteria, Firmicutes, Fusobacteria	Dietary β-(1,3)(1,6)-D-glucan supplementation impacts gut microbiota
Kim et al., 2007	*Oncorhynchus mykiss*	A	Intestinal mucosa and contents	199	16S/DGGE + Culture + Sanger Sequencing	Proteobacteria, Fusobacteria	Differences between intestinal mucosa and contents. e.g., Gut contents—Enterobacter, Bacteroides, Flavobacteria, Pasteurellacae. Mucosa =- Enterobacter, Aeromonadacae, Pseudomonadacae, Mycoplasmatacae
Silva et al., 2011	*Sparus aurata*	A	Intestinal mucosa and contents	160	16S/DGGE + Culture + Sanger Sequencing	Gamma-proteobacteria, Bacteroidetes, Firmicutes	Dominated by Photobacterium sp.
Navarrete et al., 2012	*Oncorhynchus kisutch* (juvenile)	A	Eggs + Juvenile intestinal contents	14	16S/DGGE + Sanger sequencing	Egg—Bacteriodetes (flavobacteria), Beta-proteobacteria; Juvemiles—Gamma-proteobatceria, firmicutes	–
Merrifield et al., 2013	*Dario rerio*	A	Hindgut	8	16S/DGGE + Sanger sequencing	(no order) Fusobacteria, Gammaproteobacteria	Nanoparticles included in diet disrupt communty structure
Ni et al., 2012	*Ctenopharyngodon idellus*	A/W	Intestinal mucosa and contents	75	16S/DGGE + Sanger sequencing	(no order) Cetobacterium. Aeromonas, Plesiomonas, Sporacetigenium, Enterobacter	–
Tapia-Paniagua et al., 2010	*Solea senegalensis*	A	Intestinal contents	7	16S/DGGE + Sanger sequencing	Gamma-proteobacteria	Dominated by *Vibrio* species, enhanced by prebiotics
Zhou et al., 2012	*Gadus morhua*	A	Intestinal mucosa and contents	34	16S/DGGE + Sanger sequencing	Proteobacteria, Firmicutes, Actinobacteria, Bacteriodetes, Deinococci	Genera associated with chitin-rich diet: Escherichia, Erwinia, Thermus
Geraylou et al., 2012	*Acipenser baerii*	A	Hindgut	36	16S/DGGE + Sanger Sequencing	Proteobacteria, Firmicutes, Fusobacteria	Comparison of different diets on hind gut fermentation
Liu et al., 2012	*Carassius auratus*	A	Intestinal contents	ND	16S/DGGE + Sanger Sequencing	Actinobacteria, Firmicutes, Proteobacteria	Antibiotic treatment disrupts microbiota of healthy fish more significantly than those with disease.
Li et al., 2012	*Ctenopharyngodon idellus*	A	Intestinal mucosa and contents	41	16S/DGGE + Sanger Sequencing	(no order) alpha, beta and gamma-proteobacteria, Actinobacteria	–
Li et al., 2012	*Hypophthalmichthys molitrix*	A	Intestinal mucosa and contents	41	16S/DGGE + Sanger Sequencing	(no order) Actinobacteria, Firmicutes, alpha and gamma-proteobacteria	–
Li et al., 2012	*Hypophthalmichthys nobilis*	A	Intestinal mucosa and contents	41	16S/DGGE + Sanger Sequencing	(no order) alpha, beta and gamma-proteobacteria, Actinobacteria	–
Li et al., 2012	*Megalobrama amblycephala*	A	Intestinal mucosa and contents	41	16S/DGGE + Sanger Sequencing	Beta and Gamma-proteobacteria	–
Navarrete et al., 2010	*Salmo salar*	A (marine)	Intestinal mucosa and contents	700	16S/RFLP + Sanger sequencing	Gamma-proteobacteria, Firmicutes, Bacterioidetes	Pseudomonas, Acinetobacter, Flavobacterium, Psychrobacter, Brevundimonas, Caulobacter, Mycoplana, Aeromonas, Haemophilus, Aeromonas salmonicida, Bacillus, Micrococcus/Kocuria. Reduction in diversity among tetracyclin treated indiividuals
Moran et al., 2005	*Kyphosus sydneyanus*	W	Intestinal contents	12	16S/T-RFLP, Sanger Sequencing	(no order) *Closteridium* species	Putative inviolvement in short chain fatty acid metabolism
Smriga et al., 2010	*Acanthurus nigricans*	W	Intestinal contents	48	16S/TA cloning, Sanger sequencing	Bacteriodetes, Firmicutes Proteobacteria (Vibrionacae ijn minority), Bacteriodete, Spirochaetes	–
Smriga et al., 2010	*Chlorurus sordidus*	W	Intestinal contents	44	16S/TA cloning, Sanger sequencing	Proteobacteria (mostly Vibrionacae), Bacteriodetes + other minor groups	–
Ward et al., 2009	*Chaenocephalus aceratus*	W	Intestinal contents	303	16S/TA cloning, Sanger sequencing	Gamma-proteobatceria	Photobacterium
Smriga et al., 2010	*Lutjanus bohar*	W	Intestinal contents	46	16S/TA cloning, Sanger sequencing	Proteobacteria (Vibrionacae), Firmicutes	–
Ward et al., 2009	*Notothenia coriiceps*	W	Intestinal contents	194	16S/TA cloning, Sanger sequencing	Gamma-proteobatceria	Photobacterium/Vibrio
Green et al., 2013	*Salmo salar*	A (marine)	Intestinal contents	30	16S/TA cloning, Sanger sequencing	(no order) Proteobacteria, Actinobacteria, Bacteroidetes, Firmicutes and Verrucomicrobi	Addition of soyabean derived protein resulted in dysbiotic changes in intestinal microbiota and presence of genera not normally associated with the marine environment
Larsen et al., 2013	*Cynoscion arenarius,*	W	Skin mucosa	69	16S/TA cloning, Sanger sequencing	Proteobacteria, Firmicutes, Bacteriodetes	Fish species, capture locality and capture date all influence skin microbiota
Larsen et al., 2013	*Cynoscion nebulosus*	W	Skin mucosa	69	16S/TA cloning, Sanger sequencing	Proteobacteria, Firmicutes, Bacteriodetes	Fish species, capture locality and capture date all influence skin microbiota
Larsen et al., 2013	*Lagodon rhomboides*	W	Skin mucosa	69	16S/TA cloning, Sanger sequencing	Proteobacteria, Firmicutes, Actinobacteria	Fish species, capture locality and capture date all influence skin microbiota
Larsen et al., 2013	*Lutjanus campechanus*	W	Skin mucosa	69	16S/TA cloning, Sanger sequencing	Proteobacteria, Firmicutes, Actinobacteria, Bacteriodetes, Cyanobacteria	Fish species, capture locality and capture date all influence skin microbiota
Larsen et al., 2013	*Micropogonias undulatus*	W	Skin mucosa	69	16S/TA cloning, Sanger sequencing	Proteobacteria, Firmicutes, Cyanobacteria, Actinobacteria, Bacteriodetes	Fish species, capture locality and capture date all influence skin microbiota
Larsen et al., 2013	*Mugil cephalus*	W	Skin mucosa	69	16S/TA cloning, Sanger sequencing	Proteobacteria, Firmicutes, Actinobacteria, Bacteriodetes, Cyanobacteria	Fish species, capture locality and capture date all influence skin microbiota
Navarrete et al., 2009	*Salmo salar* (juvenile)	A	Intestinal mucosa and contents	80	16S&ITS/TTGE and Sanger sequencing	Proteobacteria	Differences between gut compartments by TGGE
Arias et al., 2013	*Lutjanus campechanus*	W	Anterior Kidney	43	16S/Culture + Sanger Sequencing	Proteobacteria, Firmicutes, Actinobacteria	Firmicultes and Actinobatceria more common on the skin than in the kidney
Ringø et al., 2006	*Gadus morhua*	A	Intestinal mucosa and contents	425	16S/Culture + Sanger Sequencing	(no order) Firmicutes, Bacteriodetes, Actinobacteria, Proteobacteria	Dietary differences in microbiota. Bacteriodetes preferentially adherent. Anthrobacter absent from foregut
Arias et al., 2013	*Lutjanus campechanus*	W	Skin mucosa	179	16S/Culture + Sanger Sequencing	Proteobacteria, Firmicutes, Actinobacteria	Firmicultes and Actinobatceria are more common on the skin than in the kidney
Mansfield et al., 2010	*Oncorhynchus mykiss*	A	Ground intestinal tissue	3357	HSP60/Sanger clones libraries	Firmicutes, gamma-proteobacteria, alpha-proteobacteria, actinobacteria	–
Boutin et al., 2013a	*Salvelinus fontinalis*	A	Skin mucosa	117,260	16S/454 pyroseqeuncing	Proteobacteria (Alpha, Gamma, Beta and Delta), Actinobacteria, Bacteroidetes, Firmicutes, TM7, Chlorobi	Probiotic treatment by an indigenous strain does not disturb the natural microbiota of *Salvelinus fontinalis*
Boutin et al., 2014	*Salvelinus fontinalis*	A	Skin mucosa	87,940	16S/454 pyroseqeuncing	Proteobacteria (Alpha, Gamma), Bacteroidetes	*Salvelinus fontinalis* presents three QTL region linked to the abundance of three commensal genera
Boutin et al., 2013b	*Salvelinus fontinalis*	A	Skin mucosa	678,211	16S/454 pyroseqeuncing	Proteobacteria (Beta, Alpha, Gamma), Actinobacteria, Bacteroidetes	Host’ stress influences the skin microbiota. Commensals strains abundance decreases and favors growth of opportunistic pathogens

a *Aquaculture, A; Wild, W*.

In humans, our burgeoning understanding of our “second genome” is driving research into disease, nutrition, lifestyle, as well as immunity and development, (e.g., Furusawa et al., 2013). The applications of an improved understanding in terms of biomarkers, modulation of dysbiotic microbiomes with pre- and pro- biotics, treatment of infectious disease, as well as the generation of totally artificial microbiomes, are considerable. In teleosts, and especially in aquaculture, these applications are equally, if not more, important. Multiple phenomena could be potentially addressed through microbiome manipulation: nutrient digestion, synthesis, absorption, pathogen resistance, growth, sexual maturation, morphogenesis, survivorship in stocked fish, to name a few. In this review we asses the status-quo of teleost microbiome research with special reference to research applications in aquaculture.

## Teleost microbiomes in health and disease

### Teleost microbiomes as biomarkers for stress

Aquaculture is a growing industry. Average annual per-capita consumption of fish increased from 12.6 kg in the 1980s to 17.0 kg in 2007, meanwhile wild fish stocks are in steep decline (FAO, 2010). Unfortunately, the growing demand for fish has resulted in an intensification that impacts the welfare of animals in aquaculture systems (Ashley, 2007). Fish welfare in aquaculture may be measured via several physiological and behavioral proxies. These proxies can be usefully combined under the phenomenon of stress. The notion of stress in aquaculture is described by Barton and Iwama (1991) as a normal adaptive physiological response to overcome a negative environmental stimulus or disturbance (Barton and Iwama, 1991). In practice stressful stimuli have multiple sources—handling, sorting, grading, transport and stocking, for example. When such stimuli promote a prolonged stress response, the response may be considered maladaptive as the stress becomes detrimental to fish health.

**Figure 2:**
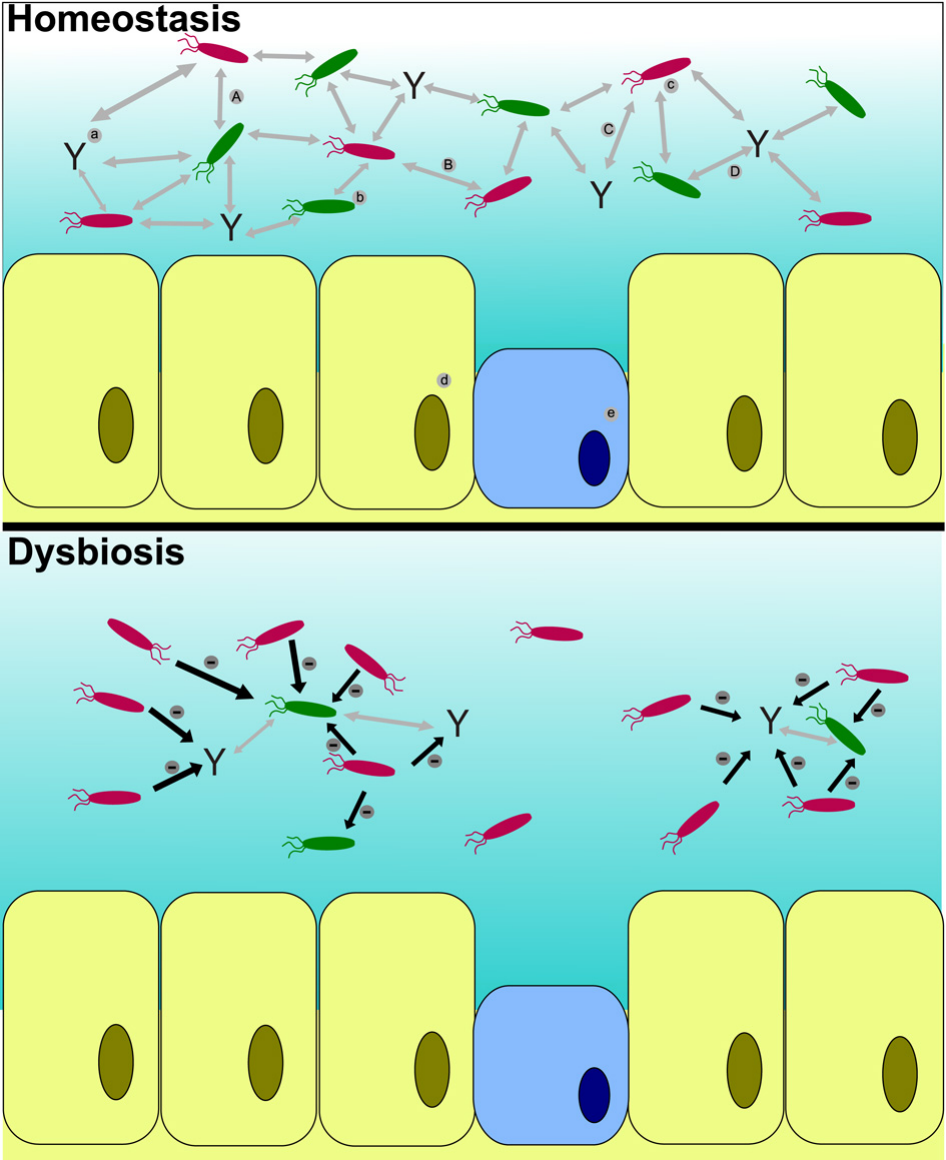
**Host microbiota interactions during homeostasis and dysbiosis**. The host is able to control the pathogen (c) growth by different process (A,C) involving the immune response (a) and the resident microbiota (b). Furthermore, the immune response recognizes the resident microbiota (D) as non-pathogenic bacteria. Pathogenic bacteria auto-regulate abundance via quorum sensing (B) and can detect environmental signals from host cells [epidermic cells (d) and mucous cells (e)]. During dysbiosis, the pathogenic population, triggered by the stress response of the host (diminution of the immune response, production of mucus and diminution of the abundance of the resident microbiota), overcome the immune response and outcompete the resident microbiota.

Microbiome balance is known to be key to maintaining overall health in fish (Gómez and Balcázar, 2008). Stress can influence the microbiome in different ways with repercussions for physiological, hormonal and cellular function. The response of the teleost epidermal mucosa to stress is associated with mucus protein compositional shift (Wendelaar Bonga, 1997; Easy and Ross, 2009; Rakers et al., 2010). The composition of the mucosa in turn shapes their microbial community, and there is evidence that stress impacts microbiome diversity in *Salvelinus fontinalis* (Boutin et al., 2013b). Network analysis of bacterial taxa present in the epidermal mucous of this salmonid during a period of prolonged artificial hypoxic stress revealed interactions between multiple bacterial players in the microbiome. Two taxonomic consortia (co-occuring taxa) emerged (Boutin et al., 2013b). The first consortium, found on unstressed control fish, comprised species from genera *Sphingomonas, Methylobacterium, Propionibacterium*, and *Thiobacter*, some of which are associated with pro-biotic and/or anti-microbial activity. The second consortium, found on stressed individuals, contained an array of different putative pathogens from the genera *Psychrobacter, Steroidobacter, Pseudomonas, Acinetobacter*, and *Aeromonas*. A conceptual overview of microbiome disruption (or “dysbiosis” as it is often termed) in the epidermal mucous of a teleost is presented in Figure 2.

At the most basic level, microbiome homeostasis (stability) is thought to be under the control of constitutively molecules and receptors of the innate immune system (Dixon et al., 2004). Stress is known to impact immunity in several teleost species (Barton and Iwama, 1991; Iger et al., 1995; Espelid et al., 1996). Teleosts share many components of innate immunity with mammals (Magnadottir, 2006). It seems likely that microbiome shifts in response to stress to an extent reflect a shift in host pattern recognition pathways. Thus, indigenous microbiota represent a valuable extension to the standard behavioral and physiological markers of stress. As we will see, stress related imbalance in the microbiome could be a precursor to disease, and thus of crucial practical importance in aquaculture.

### Teleost microbiomes in communicable disease

The immune system and commensal microbiome are though to form an integrative system of defense from communicable disease (Kitano and Oda, 2006). This system operates on two levels. Firstly, there is now good evidence that the presence of commensal bacteria facilitates the development of the vertebrate adaptive immune system (Rakoff-Nahoum et al., 2004; Kelly et al., 2005; Mazmanian and Kasper, 2006; O'Mahony et al., 2008; Hooper et al., 2012). Furthermore, the commensal microbiome inhibits colonization by pathogenic bacteria either passively, via competitive exclusion, or actively, via toxic secondary metabolites. This effect is termed “colonization resistance” (Wells et al., 1988; Balcazar et al., 2006; Stecher and Hardt, 2008). Any disturbance to the commensal microbiome, which results in dysbiosis, can thus enhance susceptibility to disease (Figure 2).

Bacterial pathogens that infect teleosts are found across multiple genera including members of Vibrio, Streptococcus, Aeromonas, Flavobacterium, Photobacterium, Pasteurella, Tenacibacterium, Pseudomonas, Lactococcus, Edwarsiella, Yersinia, Renibacterium, and Mycobacterium (Austin and Austin, 2007). Most of these organisms can survive as well as (in some cases) replicate outside their host in the aquatic milieu. In addition they are almost all opportunistic pathogens (Austin and Austin, 2007). Culture and direct sequencing based surveys of commensal skin and intestinal microbiota suggest bacterial pathogens frequently occur as a minor component of healthy teleost microbiomes but emerge as pathogens under certain circumstances, e.g., (Navarrete et al., 2010; Austin and Austin, 2012; Boutin et al., 2013a,b). Stress, usually of the prolonged, maladaptive type, is perhaps the most commonly attributed as a causal factor in aquaculture disease outbreaks (Snieszko, 1974; Wakabayashi, 1991; Wendelaar Bonga, 1997; Le Moullac et al., 1998; Sudo et al., 2004; Schimel et al., 2007; Freestone et al., 2008; O'Mahony et al., 2009; Thurber et al., 2009; Littman et al., 2010; Boutin et al., 2012; Verbrugghe et al., 2012; Moloney et al., 2013). The link between stress and disease is not limited to bacterial pathogens and teleost aquaculture. White spot syndrome virus (WSSV), for example, a major pathogen in shrimp, is commonly found in healthy populations as a commensal agent, yet the mechanisms for this latency are not clearly understood (Sanchez-Paz, 2010).

As aquaculture intensifies, host population densities have increased to support the kind of virulence shifts associated with pathogenic agents that cause large, horizontally transmitted outbreaks (Pulkkinen et al., 2010). Stress-induced microbiome dysbiosis may be a useful predictor for the emergence of opportunistic disease. However, it is not clear to what extent a healthy microbiome will protect against the more virulent aquaculture pathogens of the future. Furthermore, it remains to be seen what role the teleost microbiomes have in defining susceptibility to important ectoparasites in aquaculture (Caligidae, Monogea, etc.), as well as to the secondary bacterial infections they precipitate.

### Diet and the teleost microbiome

Most published work on teleost-associated microbiota focuses on the intestinal microbiome (Table 1). Among those experimental studies undertaken, a common line of investigation is the influence of diet on bacterial community composition. Non-marine protein supplementation is a key issue with respect to the aquaculture of predatory marine teleosts. For both *Salmo salar* and *Gadus morhua* supplementation with soya bean derived proteins resulted in significant shifts in intestinal microbiota, including the presence of bacteria atypical to marine environments (Ringø et al., 2006; Green et al., 2013). It is not clear whether these changes may be termed “dysbiotic” as the authors suggest, partly because so little is known about the “natural” state of gut microbiomes in these species. Fortunately recent work has probed the natural diversity of gut microbiota in wild Norweigian cod (*G. morhua*) via Roche 454 pyrosequencing (Star et al., 2013). The study revealed substantial inter-individual variation and suggested a predominance of Vibrionacae (proteobacteria) among the 15+ bacterial orders identified. Meaningful comparison between this dataset and previous, culture based surveys of microbiota in *G. morhua* are essentially impossible, although proteobacteria were been identified using both isolation techniques (Ringø et al., 2006; Zhou et al., 2012). The current technological shift from culture-based isolation and Sanger sequencing to direct PCR amplification and massively parallel sequencing means that meaningful comparisons are thin on the ground. The total number of bacterial sequences derived from *G. morhua* intestinal microbiomes was 459 prior to Star et al. (2013) (Ringø et al., 2006; Zhou et al., 2012; Star et al., 2013). The pyrosequencing Star et al. (2013) undertook increased this tally by 280,447.

Whether or not teleost microbial studies have used the most up-to-date methods for profiling gut bacterial communities, the themes on which they touch are certainly valid, and form a platform for future research. As well investigating the impact of soya protein, researchers have evaluated the impact of dietary chitin on the microbiome (Zhou et al., 2012). Chitin represents a huge, but largely indigestible, potential source of carbohydrates for fish. It is of considerable interest what role indigenous gut microbiota might play in chitin decomposition. Similarly, the presence of cellulolytic microbial species in the intestines of the wood eating catfish has been probed (Di Maiuta et al., 2013). Such studies will benefit from functional characterization of the bacterial metagenetic repertoire, and teleost alimentary tracts promise rich veins for glycide hydrolase bioprospecting, given the huge variety of different dietary niches they exploit.

## Microbiome manipulation

### Probiotics

It is understood that several parameters: genetic, nutritional and environmental; affect the abundance and diversity of gut microbiota in fish (Dimitroglou et al., 2011; Daniels and Hoseinifar, 2014; Ringø et al., 2014). The idea of manipulating gut microbiota of fish developed as a consequence of the fact that potentially beneficial bacterial communities such as lactic acid bacteria naturally constitute only a minor proportion of intestinal microbiota of fish or shellfish (Ringø et al., 2010). It has been suggested that the manipulation of fish gut microbiota will result in elevation of resistance against pathogens, growth enhancement, improved lipid metabolism, stimulation of immune response and better physiological status for the gut (Tellez et al., 2006). Thus, there is increasing interest in strategies for the manipulation of gut microbiota of fish toward beneficial communities (e.g., lactic acid bacteria) (Daniels and Hoseinifar, 2014; Ringø et al., 2014).

A primary approach toward microbiome manipulations is the administration of probiotics, which are defined as live microbial culture added to feed or environment (water) to increase viability (survival) of the host (Gram and Ringø, 2005). This definition is being constantly refined and updated associated with health promoting properties (Irianto and Austin, 2002a) or with other benefits. The latest accepted definition for probiotics for aquatic animals is suggested by Merrifield et al. (2010). According to the authors probiotic for aquaculture is a live, dead or component of a microbial cell that, when administered via the feed or to the rearing water, benefits the host by improving either disease resistance, health status, growth performance, feed utilization, stress response, which is achieved at least in part via improving the hosts or the environmental microbial balance.

Although the mechanisms by which probiotics exert their beneficial effects on the host are largely unknown, probiotics administration showed promising results on growth performance and health of teleost fish (Gatesoupe, 2010). Despite the aforementioned advantages of probiotics, the viability of live bacteria during large-scale production of food (i.e., commercial diets) and during transition through the gastrointestinal tract is not reliable (Ringø et al., 2014).

### Prebiotics

To resolve issues with probiotics, the prebiotic concept has been suggested and developed (Mahious and Ollevier, 2005). A prebiotic is a non digestible food ingredient that beneficially affects the host by selectively stimulating the growth and/or activity of one or a limited number of bacteria in the colon, that can improve the host health (Roberfroid, 2007). According to Gibson (2004) a dietary ingredient should meet the following criteria to be classified as a prebiotic, (1) resist gastric acidity, hydrolysis by digestive enzymes and gastrointestinal absorption; (2) be fermented by the intestinal microbiota and; (3) be able to selectively stimulate the growth and activity of beneficial bacteria (Gibson, 2004). To our knowledge the first study on prebiotics in aquaculture was reported by Hanley et al. (1995). Since then the most common prebiotics studied in fish were inulin, fructooligosaccharides (FOS), short-chain fructooligosaccharides (scFOS), mannanoligosaccharides (MOS), *trans*-galacto-oligosaccharides (TOS), Bio-MOS® containing MOS derived from yeast, galacto-oligosaccharides (GOS), xylooligosaccharides (XOS), arabinoxylooligosaccharides (AXOS), isomaltooligosaccharides (IMO), GroBiotic®-A (GBA) (Ringø et al., 2014). Beneficial bacterial members of the gut microbiota use prebiotics as substrate for growth. 454 pyrosequencing has recently confirmed this effect in juvenile Siberian sturgeon (*Acipenser baerii*) fed with an AXOS prebiotic (Geraylou et al., 2012). In this work, significant increases in abundance of several bacterial families, including *Lactobacillaceae*, were observed in individuals with AXOS treatment regimes. Another important product of prebiotic fermentation by gut microbiota is short chain fatty acid (SCFA) (Cummings and Macfarlane, 2002). SCFA are the main energy source for colonic epithelial cells and thus associated with maintenance of the epithelium (Maslowski and Mackay, 2010). Moreover, it has been proposed that SCFA modulates lipid synthesis (Marcil et al., 2002) and has the potential to stimulate the immune system and resistance against pathogens (Maslowski and Mackay, 2010). However, it remains to be seen precisely which microbial taxa play a dominant role in SCFA production in fish.

### Synbiotics

A recent concept in regards to the manipulation of gut microbiota are synbiotics. Synbiotics refer to nutritional supplements combining probiotics and necessary nutrients for their survival (Cerezuela et al., 2011). As such, synbiotics aim to simultaneously seed and maintain probiotic strains as the dominant species in the gut after treatment cessation (Rurangwa et al., 2009). Despite recent progress in the field of synbiotics administration in aquaculture, there is limited information available on different aspects of synbiotics' effects on fish (Cerezuela et al., 2011).

### Probiotics and disease

The use of probiotics as biological control agents for disease is fairly well established in aquaculture, in contrast to other areas of animal and human health, where it seems all but absent as an approach (Newaj-Fyzul et al., 2013). Bacterial cultivars from over 30 different genera are have been administered (Newaj-Fyzul et al., 2013). Target disease agents are usually bacterial, and infection with a wide variety of pathogens has been treated in several different teleost species, primarily in aquaculture. *Aeromonas hydrophila* has been successfully used *in vivo* to treat *A. salmonicida* infection in *Oncorhynchus mykiss*, for example (Irianto and Austin, 2002b). Meanwhile *Rhodococcus qingshengii* had been successfully applied to the treatment of *Flavobacterium psychrophilum* infection in *Salevinus fontinalis* (Boutin et al., 2012). There are numerous examples in the literature of such trials (Newaj-Fyzul et al., 2013), however, it is by no means clear by what mode of action these agents operate, especially in the context of the wider microbiome. While some effective probiotics, particularly those administered prior to challenge with the infectious agents, (e.g., De la Banda et al., 2012), may to an extent bolster the “colonization resistance” of the indigenous microbiome, the action of others is less clear still. Longitudinal surveys of the indigenous microbiome during these trials are sparse, and there is clearly significant scope for further research.

### Host genetics and teleost microbiomes

The level of influence that host genome exerts on microbiome composition is a matter for debate, even in well-studied organisms like humans (Spor et al., 2011). There is evidence that the quantitive trait loci (QTL) can detect an influence of host genetic variation on fecal microbiome composition in mice (Benson et al., 2010). Those taxa under host genetic control corresponded with species and genera thought to interact with host immunity (Benson et al., 2010). QTL analysis of skin microbiome composition has recently been undertaken in the salmonid *Salvelinus fontinalis* (Boutin et al., 2014) and “common garden experiments” on different *O. mykiss* families have also explored associations with host background (Navarrete et al., 2012). As with mice, in both cases there is some limited evidence for host genetic control. At the inter-species level, there may some level of host-specificity in teleost larvae as well (Li et al., 2012). Given that maternal effects can be largely discounted in fish, the mechanism through which such control is exerted must be innate immunity. Pathogen Recognition Receptors (PRRs)—comprised of Toll-like receptors (TLRs), and their co-receptor CD14, the scavengers receptors, the mannose receptors, the integrins CD11b-c/CD18 and the complement receptors CR1,2,3—form a major component in innate immunity. PRRs are expressed at the surface of the cells to recognize a variety of non-host ligands collectively termed microbe associated molecular patterns (MAMPs) (Medzhitov and Janeway, 1999). Standing genetic variation among components of the teleost adaptive immune system is increasingly well characterized, (e.g., Dionne et al., 2009; Pavey et al., 2013). While TLRs are present in multiple teleost species (Palti, 2011), there has been no work to date to correlate genetic diversity at these innate immune loci (inter- or intra- species) with commensal microbiome diversity. Experiments in zebrafish highlight the role that TLRs play in modulating intestinal microbiota, whereby alkaline phosphatase is produced via a TLR-4-myD88 controlled pathway to inhibit an inflammatory responses to gut microbiota (Bates et al., 2007). Given that desirable microbiome characteristics from an aquaculture perspective may exist (e.g., disease resistance, nutrient absorption, stress resilience), it is encouraging that a host genetic basis may exist to select for such traits.

### Microbiome ontogenesis

Intensive aquaculture is hampered by unpredictable mortalities during early life stages that are likely due, at least in part, to negative interactions between fish larvae and some bacterial strains they routinely encounter. In order to control mortalities at early life stages, the aquaculture industry prioritized egg and larvae disinfection protocols. Such guidelines are perhaps counter-productive, given that most of the bacteria routinely isolated from hatcheries are not harmful to larvae (Verner-Jeffreys et al., 2003), and fish microbiota are the first line of defense against pathogens (Boutin et al., 2012).

**Figure 3:**
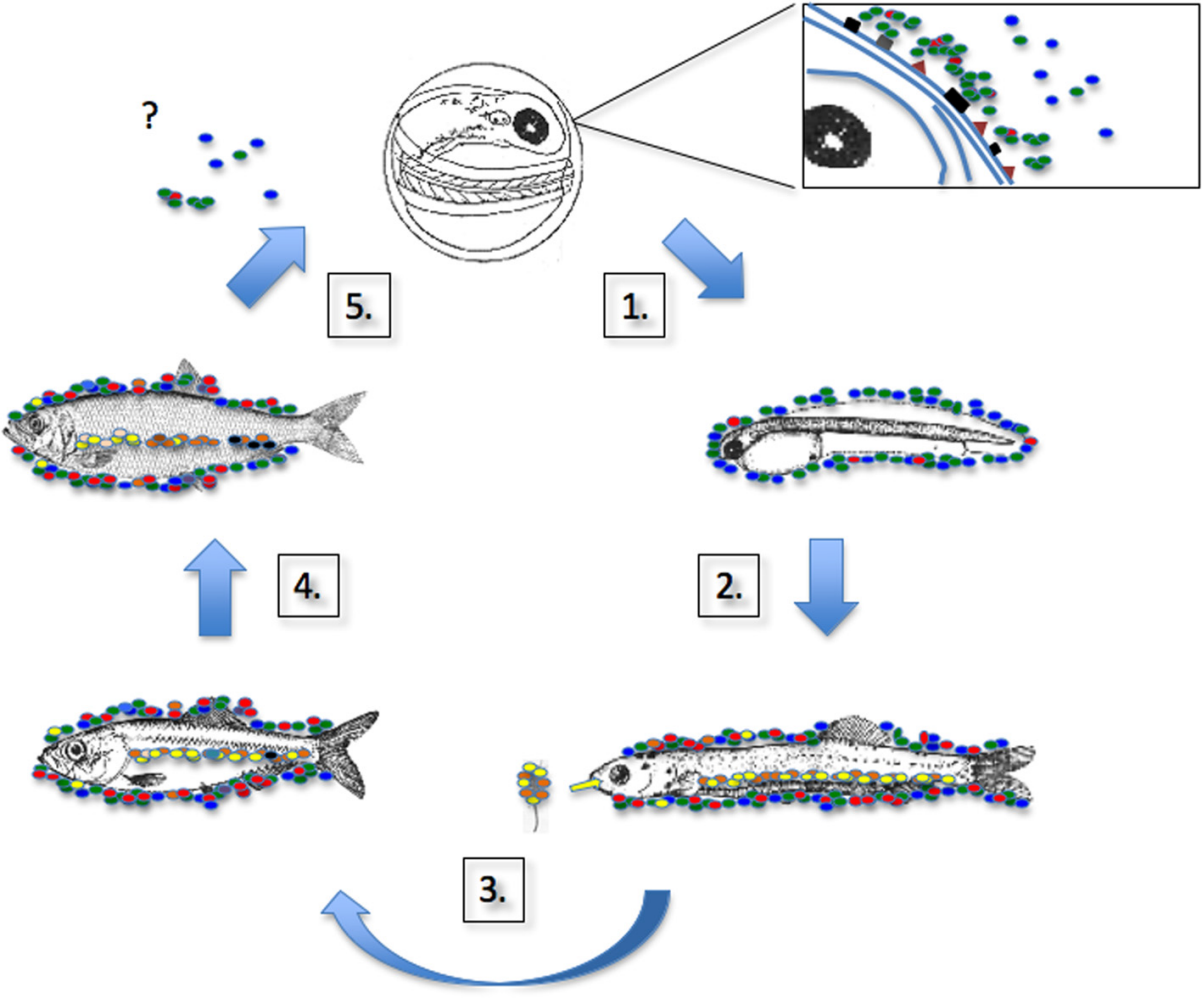
**Teleost microbiome during development**. Figure shows schematic of the generalized lifecycle of a teleost and accessory indigenous bacteria (different taxa represented by colored elipses). (1) Bacteria colonize the chorion of the egg. Taxonomic differences of bacteria between fish species suggest specific early interactions, perhaps through precursors of innate immunity (symbolized by squares and triangles on the chorion surface). (2) Egg hatches, larval is colonized by environmental bacteria as well as those originally present on the chorion. (3) Early digestive tract colonization occurs when larva commence feeding. Bacterial taxa strongly resemble those associated with food source. (4) Microbiome develops, accumulates diversity and matures. (5) Adult microbiome is diverse assemblage of microbial taxa. Differences exist between surface mucosal and intestinal communities. Intestinal communities also be compartmentalized/specialized to niches within the alimentary tract. Question mark indicates possible vertical transmission of microbiome components to eggs during oviposition.

Early promotion of nutrient metabolism and innate immune response depend upon the bacterial species that colonize the digestive tract. It is therefore of primary importance to understand the mechanisms that orchestrate the early steps of colonization of the gastrointestinal tract of fish, leading the buildup of a stable, diversified and resilient endogenous microbial community. Colonization steps are summarized in Figure 3.

Culture-based identification of bacterial species has been the mainstay of studies examining early teleost microbiome development to date, but their finding are nonetheless intriguing. In the aquatic environment, bacteria move easily between habitats and hosts. Thus the first steps of interactions and colonization of fish progeny occur as soon as the eggs are laid. The number of bacteria colonizing salmonid eggs, for example, ranges between 10^3^ and 10^6^ bacteria g^−1^ (Yoshimizu et al., 1980). The diverse microbiota that eventually develops on the egg surface is expected to reflect the bacterial composition of the water. Interestingly, species-specific differences were observed in terms of bacterial colonization of fish eggs between cod and halibut (Hansen and Olafsen, 1989). Such host specific assemblages on the chorion may result from differential attraction to surface receptors, to those being coded by host genotype. Once eggs hatch, sterile larvae are rapidly colonized by ova debris and microbiota present in the environment (Hansen and Olafsen, 1989). Passage of surface bacteria into the gut is expected to colonize larvae gut as soon they are begin to ingest their liquid medium (Lauzon et al., 2010). Unsurprisingly, the alimentary tract of first-feeding fries is colonized with bacteria associated with food (Blanch et al., 1997; Korsnes et al., 2006; Reid et al., 2009). The process of recruitment of taxa to the developing microbiome clearly has to work with those bacteria present in the immediate environment.

Romero and Navarrete (2006) pioneered the identification of dominant bacterial populations associated with early life stages of salmon coho using a 16S RNA barcoding approach using a DGGE metagenomic (culture-independent) approach (Romero and Navarrete, 2006). They focused on three developmental stages (eggs, first-feeding fry, juvenile) and documented environmental bacterial communities (surrounding water, pelletized feed) in order to determine the putative origin of dominant intestine tract strains. Interestingly, a dominant *Pseudomonas* sp. found in the juvenile gastrointestinal tract was also present on eggs, but not in the water nor in food. This may suggests a vertical transmission of a pioneering strain, which is commonly observed as a dominant genus in gut microbiota of mature fish (Hansen and Olafsen, 1999; Jensen et al., 2004; Navarrete et al., 2010). Overall, DGGE profiles showed pioneering communities harboring very few ribotypes, those encountered important shifts, at least in terms of taxonomic diversity, between eggs, first-feeding fry, and juvenile step. The authors concluded that the early steps of the gut microbiota colonization by bacterial strains do not reflect a stable microbiota, which would be established after the first feeding stages, by recruiting its major components from water and prey epibiota. Such finding corroborates the observation that during the initial stage of gut colonization, microbiota is highly unstable in humans (Palmer et al., 2007; Mariat et al., 2009; Cho and Blaser, 2012) and mice (El Aidy et al., 2012, 2013). Furthermore, the temporal pattern in which gut microbiota evolves is characterized by a remarkable interindividual variation. Over time, microbial groups that typically dominate the adult intestinal microbiota overcome the early-colonizing microbes that are less adapted to the intestinal environment (Palmer et al., 2007; El Aidy et al., 2013).

Because the early stages of fish development are the most sensitive regarding to outbreak caused by opportunistic pathogens, and because fish microbiota are now understood as the very first barrier against opportunistic pathogens, it is of primary importance to identify the factors that control the early steps of colonization of the fish microbiota, in order to maximize the rearing conditions leading to the buildup of a stable, diversified and resilient endogenous microbial community. Gnotobiotic models starting with germ-free larvae provide an excellent tool to disentangle accurately the host microbe interactions (Rawls et al., 2004, 2006; Dierckens et al., 2009; Rekecki et al., 2013; Rendueles et al., 2013). For example zebrafish (*Danio rerio*), a widely used cyprinid fish as a valuable vertebrate developmental model, proved to be convenient for studying gut microbiota ontogenesis, host-microbiota and host-pathogen interactions (Rawls et al., 2004, 2006; Kanther, 2010). Thus, far, more than 20 pathogenic strains have been tested on germ free zebrafish (van der Sar et al., 2004; Lesley and Ramakrishnan, 2008; Kanther, 2010; Kanwal et al., 2013) or colonized with an artificial microbiota (Rawls et al., 2006; Cheesman and Guillemin, 2007; Kanther et al., 2011). Similar experiments were undertaken in non-model fish such as cod (Forberg et al., 2012), sea bass (Rekecki et al., 2013), and halibut (Verner-Jeffreys et al., 2003). In general, the results from most studies involving challenge of wild type or germ-free fish larvae with opportunistic pathogens highlight the protective role of the indigenous bacteria (Kanwal et al., 2013; Rendueles et al., 2013).

## Conclusions

At the time of writing, teleost microbiome research is on the cusp of significant progress. Next generation sequencing is increasable affordable, computationally achievable in small laboratories, and generally accessible to the wider scientific community outside model vertebrates. In this review we have highlighted areas of current interest for teleost microbiome research, namely as biomarkers for stress and disease resistance. Diet is also a major area for microbiome research, especially with regards to new feed sources to mitigate the environmental impact of aquaculture. We discussed current approaches to directly manipulate host microbiomes via pro-, pre- and synbiotics in an attempt to improve fish condition and treat disease, as well as a host genetic basis for microbiome diversity, which could be used to select for desirable microbiome traits in the future. Finally we touched on microbiome ontogenisis in juvenile fish, crucial for the development healthy digestion and immunity.

Whilst the areas of research interest have largely been defined, the technology will shortly shift. Once next generation sequencing is routinely used to characterize teleost microbiomes, it should become significantly easier to make meaningful comparisons between species, studies, research centers and sample sites. In line with approaches defined by the HMP (Human Microbiome Project, 2012), it is extremely important to establish baselines for natural teleost microbiomes before meaningful conclusions can be drawn from the same species in aquaculture. The term “dysbiosis” is currently over-used given that the natural stability (or instability) of any teleost microbiome is not currently known.

The next 5–10 years will be an exiting time for teleost microbiome research. The timing couldn't be better given the parlous state or many wild fish stocks, the increasing global demand for fish protein, and the need to find sustainable approaches to improve aquaculture yield and mitigate its impact on marine and freshwater environments.

### Conflict of interest statement

The authors declare that the research was conducted in the absence of any commercial or financial relationships that could be construed as a potential conflict of interest.
